# Extreme Hyperlipasemia in End-Stage Renal Disease Mimicking Acute Pancreatitis: A Diagnostic Pitfall

**DOI:** 10.7759/cureus.106974

**Published:** 2026-04-13

**Authors:** Mateen Sheikh, David Casto, Michelle Braha, Guillermo Izquierdo-Pretel

**Affiliations:** 1 Medicine, Florida International University, Herbert Wertheim College of Medicine, Miami, USA; 2 Internal Medicine, Florida International University, Herbert Wertheim College of Medicine, Miami, USA; 3 Hospital Medicine, Jackson Memorial Hospital, Miami, USA

**Keywords:** end-stage renal disease (esrd), hyperlipasemia, macroamylasemia, macrolipasemia, non-pancreatic hyperenzymemia, peritoneal dialysis

## Abstract

Marked elevations of pancreatic enzymes are commonly interpreted as evidence of acute pancreatitis. However, in patients with end-stage renal disease (ESRD), serum amylase and lipase may be elevated without pancreatic inflammation, posing a significant diagnostic challenge. We present a case illustrating the diagnostic dilemma of extreme pancreatic enzyme elevation in an ESRD patient undergoing peritoneal dialysis without clinical or radiologic evidence of pancreatitis. A 50-year-old man with ESRD on peritoneal dialysis and known gastroparesis presented with intractable nausea and vomiting. Initial laboratory testing revealed markedly elevated pancreatic enzymes, with lipase levels peaking above 4,000 U/L and serum amylase at 545 U/L, raising concern for acute pancreatitis. However, the patient lacked characteristic epigastric pain, and contrast-enhanced abdominal CT demonstrated a normal pancreas without inflammatory changes or peripancreatic fluid collections. Autoimmune pancreatitis was excluded with negative IgG4 levels. Analysis of peritoneal dialysis fluid revealed very low amylase levels (less than 30 U/L), arguing against pancreatic enzyme leakage into the peritoneal cavity. Enzyme levels gradually declined over the course of hospitalization, and polyethylene glycol precipitation testing was pursued to evaluate for macroenzyme formation. The marked discordance between extremely elevated serum pancreatic enzymes and the absence of clinical symptoms, radiologic abnormalities, or dialysate enzyme elevation strongly suggested macrolipasemia and macroamylasemia in the context of ESRD. These findings likely reflect reduced renal clearance and macroenzyme complex formation rather than true pancreatic inflammation. This case highlights that extreme elevations of pancreatic enzymes may mimic acute pancreatitis in ESRD patients despite the absence of pancreatic disease. Careful correlation of symptoms, imaging findings, dialysis fluid analysis, and targeted laboratory testing is essential to avoid misdiagnosis and unnecessary interventions. Recognition of macroenzyme-associated hyperlipasemia should be considered when evaluating dialysis patients with markedly elevated pancreatic enzymes.

## Introduction

Serum lipase and amylase are widely used biomarkers for the diagnosis of acute pancreatitis, and elevations greater than three times the upper limit of normal (ULN) are often considered diagnostic when accompanied by characteristic abdominal pain or supportive imaging [1,2]. In end-stage renal disease (ESRD), however, baseline elevations of pancreatic enzymes are common in the absence of pancreatic pathology, complicating interpretation [3,4].

In ESRD, both amylase and lipase may be elevated due to reduced renal clearance, and many asymptomatic patients demonstrate hyperamylasemia or hyperlipasemia [3,5]. Impaired glomerular filtration and tubular excretion contribute, and in peritoneal dialysis patients, the formation of macroenzyme complexes (i.e., high-molecular-weight aggregates of enzymes bound to immunoglobulins or other serum proteins) may further impair clearance and prolong circulating half-life [6,7].

Macrolipasemia and macroamylasemia are rare conditions characterized by enzyme-immunoglobulin complexes exceeding 200 kDa that prevent effective renal clearance, resulting in persistent hyperenzymemia despite the absence of pancreatic inflammation [6,8,9].

Extremely high lipase values (e.g., greater than 3-4 times ULN) usually prompt a presumptive diagnosis of acute pancreatitis and may trigger imaging, prolonged hospitalization, and inappropriate management. In patients with peritoneal dialysis, distinguishing true pancreatitis from non-pancreatic enzyme elevation requires careful integration of clinical presentation, imaging, and specialized testing, including dialysate enzyme analysis.

This case addresses a key clinical question: how should clinicians interpret extreme lipase elevations, in this instance exceeding 4,000 U/L, in dialysis patients whose symptoms and imaging do not support pancreatitis? We describe an ESRD patient in whom severe hyperlipasemia initially raised concern for acute pancreatitis, but systematic evaluation was more consistent with macrolipasemia and macroamylasemia.

## Case presentation

Clinical history

A 50-year-old man with ESRD on peritoneal dialysis since April 2025 presented to the emergency department with nausea, vomiting, and generalized weakness. His medical history included hypertension, gastroparesis, gastritis, and duodenitis. He reported a six-month history of intermittent nausea and vomiting without clear triggers, with acute worsening over the preceding one to two months. One month prior, he had been hospitalized for similar symptoms; esophagogastroduodenoscopy revealed gastritis, duodenitis, and a Mallory-Weiss tear, and a gastric emptying study confirmed gastroparesis. He was discharged on metoclopramide, ondansetron, erythromycin, and omeprazole.

Despite this regimen, he continued to experience daily bilious emesis. In the two weeks before his current admission, an outpatient gastroenterology evaluation included a discussion of gastric peroral endoscopic myotomy for refractory gastroparesis. He denied fever, chest pain, dyspnea, hematemesis, melena, or hematochezia. Importantly, he denied abdominal pain at presentation. He did not consume alcohol, tobacco, or recreational drugs.

Diagnostic evaluation

On admission, laboratory testing showed a serum lipase level of 1,328 U/L. Additional abnormalities included a creatinine of 17.3 mg/dL (baseline ESRD), an anion gap of 25 mEq/L, and a hemoglobin of 11.6 g/dL. The white blood cell count was 9.4 × 10³/μL without a left shift, and glucose was 130 mg/dL.

Despite supportive care, nausea and vomiting persisted. Later during the hospitalization, repeat lipase levels increased dramatically to more than 4,000 U/L, and serum amylase was 545 U/L. Inflammatory markers (erythrocyte sedimentation rate, C-reactive protein, ferritin, procalcitonin) were elevated, raising concern for severe acute pancreatitis or autoimmune pancreatitis. Serum IgG4 was within normal limits, ruling out autoimmune pancreatitis. Complement (C3, C4) and kappa/lambda light chain ratio were normal. Peritoneal dialysis fluid analysis showed a white blood cell count less than 1 cell/μL, not consistent with peritonitis, and an amylase less than 30 U/L. The patient’s peritoneal dialysis regimen did not include icodextrin-based solutions, minimizing potential confounding effects on serum enzyme measurements.

**Figure 1:**
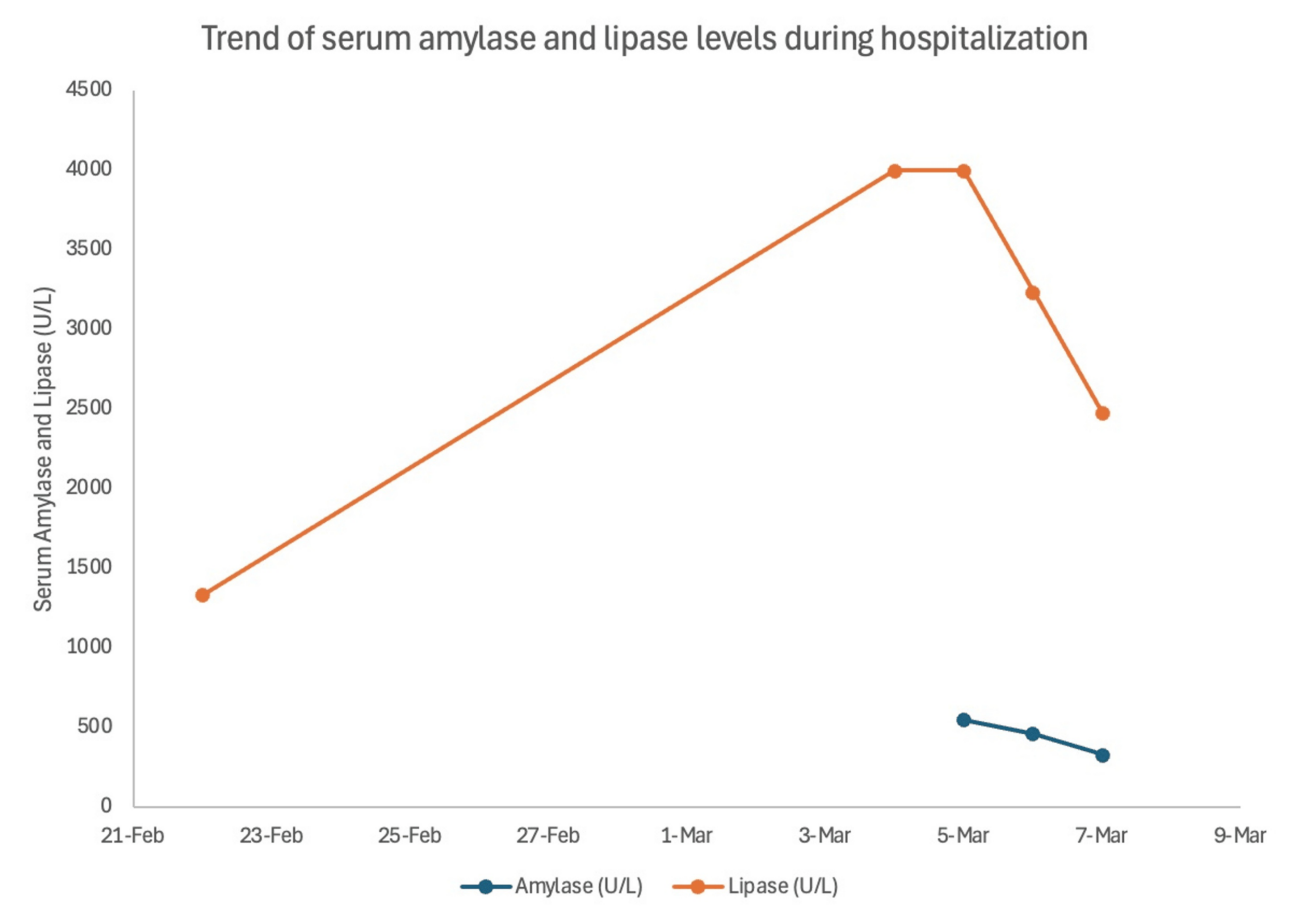
Serial serum amylase and lipase measurements from February 22 to March 7 This demonstrates marked hyperlipasemia with peak values exceeding the assay’s upper reporting limit on March 4 and March 5, recorded as 4,000 U/L on the graph. Amylase and lipase levels then gradually declined despite the absence of radiologic evidence of pancreatitis, illustrating the discordance between extreme enzyme elevation and clinical/imaging findings in this ESRD patient and supporting a diagnosis of macrolipasemia/macroamylasemia rather than acute pancreatitis. ESRD: end-stage renal disease

Serial laboratory monitoring over the subsequent days demonstrated a gradual downtrend in both lipase and amylase without pancreatic-specific interventions, as outlined in Table 1 and Figure 1 below.

**Table 1:** Pancreatic enzyme values during hospitalization Lipase peaked at >4,000 U/L despite normal CT imaging. Peritoneal dialysate amylase remained very low (<30 U/L), arguing against acute pancreatitis. CT: computed tomography

Laboratory test	Values	Reference range
Lipase	>4,000 U/L	7-60 U/L
Amylase	545 U/L	25-115 U/L
Dialysate amylase	<30 U/L	<30 U/L
Dialysate lipase	7 U/L	<10 U/L

Polyethylene glycol (PEG) precipitation testing was sent to evaluate for macrolipasemia and macroamylasemia; however, results were not available at the time of manuscript preparation because the assay is performed as a reference laboratory send-out test.

A chest radiograph on admission showed well-aerated lung bases without acute cardiopulmonary disease. Abdominal radiograph confirmed correct Tenckhoff catheter position in the right pelvis and a partially distended stomach, consistent with delayed gastric emptying, without obstruction or free air.

**Figure 2:**
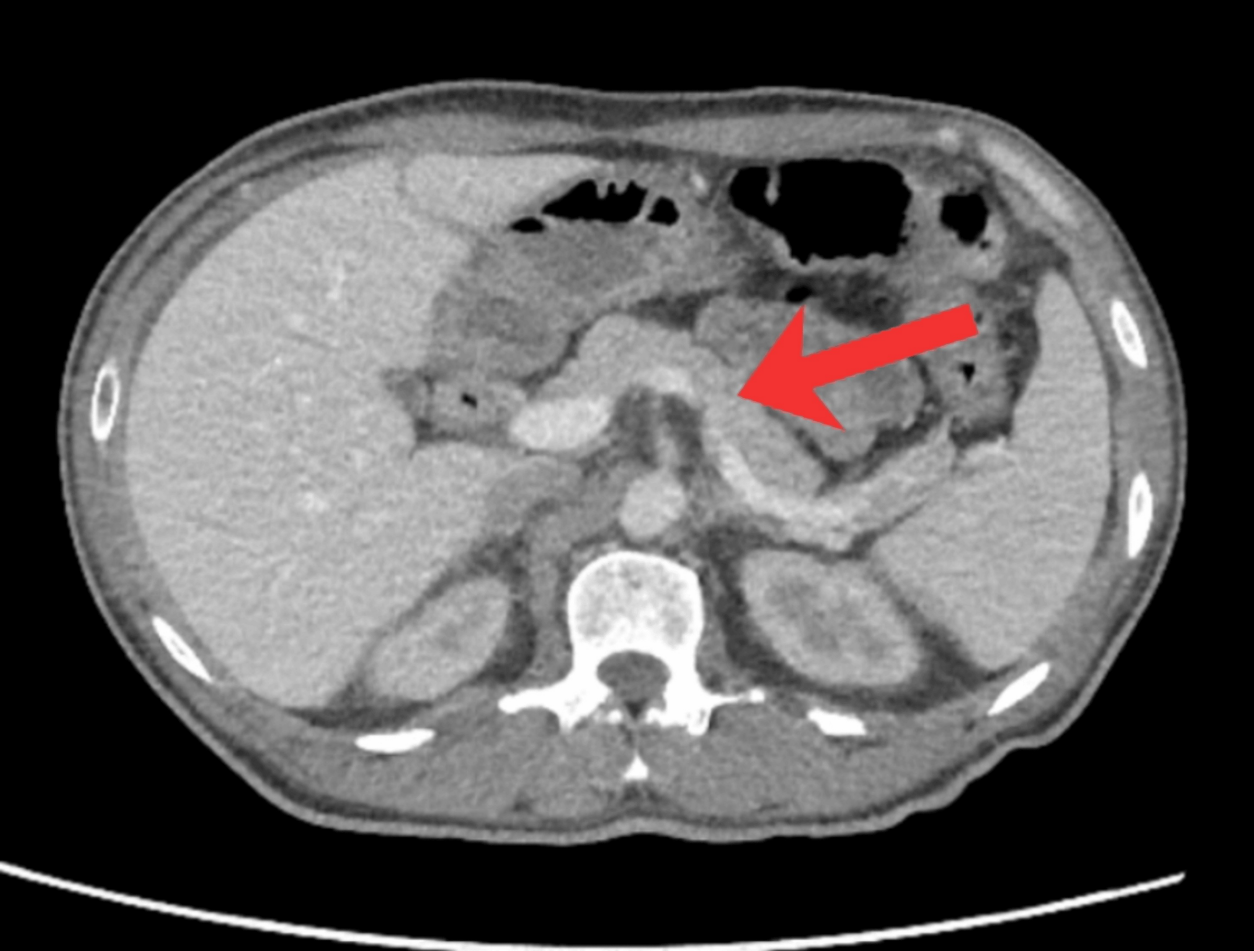
Contrast-enhanced CT of the abdomen demonstrating a normal-appearing pancreas without enlargement, peripancreatic fat stranding, fluid collections, or necrosis, despite serum lipase levels exceeding 4,000 U/L

Given the extreme lipase elevation, contrast-enhanced CT of the abdomen and pelvis was performed on hospital day 11 (March 4, 2026) to evaluate for acute or autoimmune pancreatitis. CT demonstrated a normal-appearing pancreas without inflammatory changes, edema, necrosis, or peripancreatic collections (Figure 2).

**Figure 3:**
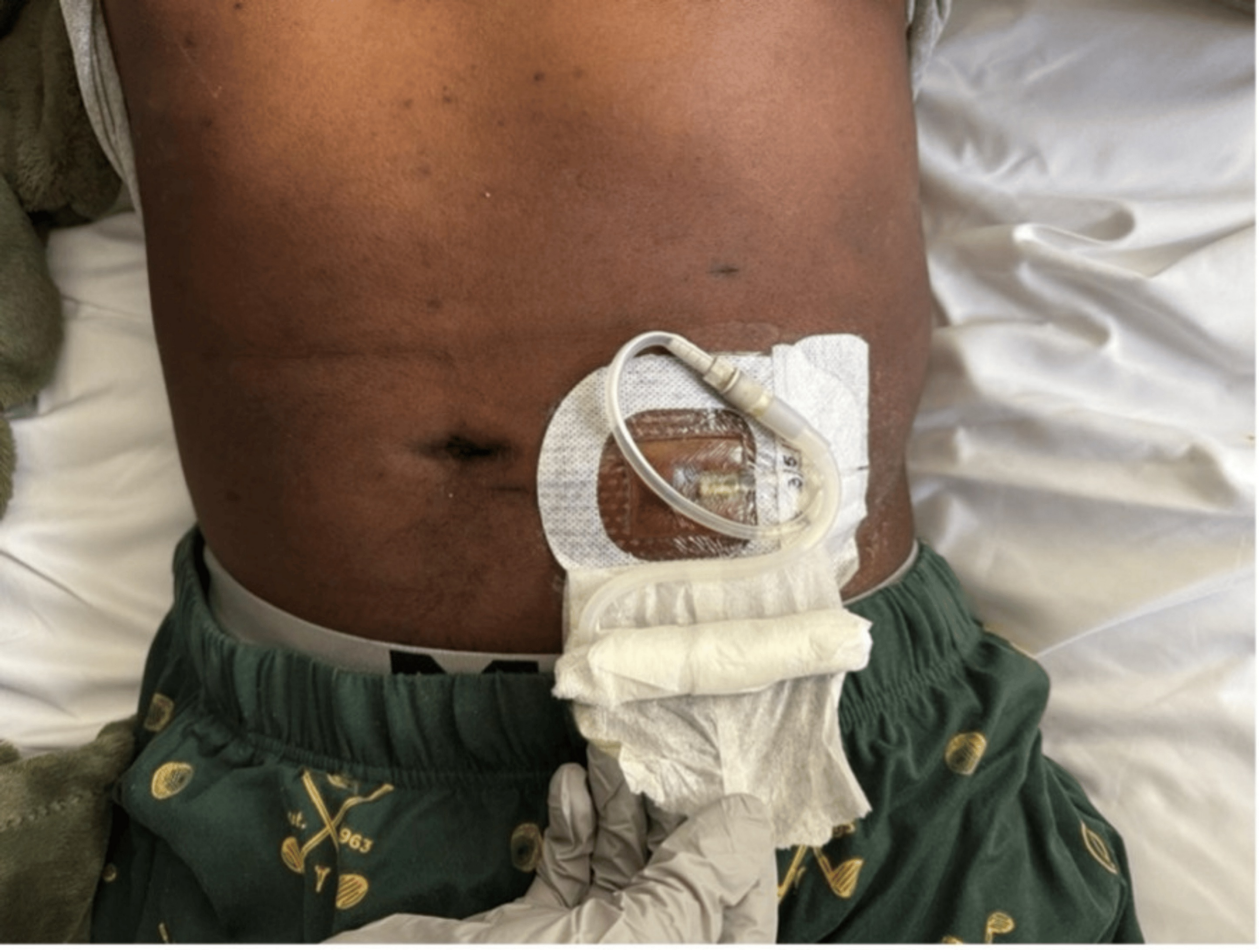
External view of the abdominal wall showing the peritoneal dialysis Tenckhoff catheter exit site with protective dressing in place The catheter is appropriately positioned in a patient undergoing chronic peritoneal dialysis. In this case, peritoneal dialysate analysis demonstrated very low amylase and lipase levels, arguing against pancreatic enzyme leakage into the peritoneal cavity and supporting the absence of acute pancreatitis despite markedly elevated serum lipase.

The peritoneal dialysis catheter was in the appropriate position (Figure 3).

Follow-up and outcomes

The patient improved gradually with supportive care directed at his known gastroparesis and possible pancreatitis, including scheduled antiemetics, prokinetic therapy, IV fluid resuscitation, and gradual advancement of oral intake. However, the patient was initially kept NPO and worked up for acute pancreatitis, given a markedly elevated lipase (peaking at ~4,000) and elevated inflammatory markers. A CT abdomen performed on hospital day 11 showed a normal pancreas without inflammatory changes, and IgG4 was negative, effectively ruling out acute and autoimmune pancreatitis. The declining enzyme levels, along with normal imaging and the absence of abdominal pain, shifted the clinical picture toward macrolipasemia/macroamylasemia as the more likely etiology of hyperenzymemia, rather than true pancreatic inflammation. This was further supported by the peritoneal dialysis fluid amylase of less than 30, arguing against pancreatic enzyme leakage into the peritoneal cavity.

PEG precipitation testing was sent out for evaluation of macroenzymemia. At discharge, the patient was clinically and hemodynamically stable, and outpatient follow-up with gastroenterology and nephrology was arranged for ongoing management of his refractory gastroparesis and ESRD on peritoneal dialysis.

## Discussion

Pancreatic enzyme metabolism in ESRD

The kidneys play a central role in the clearance of pancreatic enzymes. Amylase (~55 kDa) is primarily cleared by glomerular filtration, with an amylase-to-creatinine clearance ratio of about 2-5% in healthy individuals [10,11]. Lipase (~48 kDa) is also cleared renally, though to a lesser extent [12].

In chronic kidney disease and ESRD, reduced glomerular filtration leads to the accumulation of both amylase and lipase. A substantial proportion of asymptomatic ESRD patients have elevated pancreatic enzymes without clinical or imaging evidence of pancreatitis [3,5]. In peritoneal dialysis populations, baseline elevations of amylase are common and typically remain within 1.5-3 times ULN [3].

Mechanisms beyond reduced renal clearance likely contribute, including decreased peripheral clearance, altered enzyme degradation, possible increased pancreatic release in uremia, and the formation of macroenzyme complexes [3,6]. Dialysis does not consistently normalize serum enzyme levels, particularly for macroenzymes, because larger complexes exceed the molecular-weight cutoff of standard dialysis membranes [6].

Macroenzymes and diagnostic testing

Macroenzymes are high-molecular-weight complexes formed when circulating enzymes bind immunoglobulins or other serum proteins, resulting in molecules too large for glomerular filtration [6,8]. Macroamylasemia is the best-characterized example and is typically benign, presenting as isolated hyperamylasemia in otherwise asymptomatic individuals [6,9]. Macrolipasemia is less frequently reported but follows similar pathophysiology [6,13].

Patients with macrolipasemia or macroamylasemia often exhibit persistently elevated enzyme levels, sometimes several-fold above ULN, but without characteristic abdominal pain or imaging abnormalities [6,9,13]. This is in contrast to acute pancreatitis, which presents with acute epigastric pain, clinical deterioration, and characteristic imaging changes [1,2]. Key discriminating features between acute pancreatitis and macrolipasemia/ESRD hyperenzymemia are outlined in Table 2.

**Table 2:** Diagnostic features distinguishing acute pancreatitis from macrolipasemia CT: computed tomography, ULN: upper limit of normal, PD: peritoneal dialysis, PEG: polyethylene glycol

Feature	Acute pancreatitis	Macrolipasemia
Abdominal pain	Present (epigastric)	Absent
Nausea/vomiting	Present	Usually absent or mild
CT pancreas	Abnormal (edema, fluid)	Normal
Serum lipase	>3× ULN	Elevated, persistent
Dialysate amylase (PD)	Very high (>500-1000 U/L)	Very low (<30-50 U/L)
Symptom onset	Acute	Chronic/persistent
Enzyme trend	Rapid rise and fall	Persistently elevated
PEG precipitation	Normal (<60%)	Elevated (>60%)

PEG precipitation is the most practical first-line screening test for macroenzymes [6]. PEG selectively precipitates high-molecular-weight proteins, including immunoglobulins and macroenzyme complexes. Enzyme activity is measured before and after PEG treatment, and the percentage of activity lost (PEG-precipitable activity) is calculated; a high percentage loss suggests that a large fraction of circulating activity resides in a high-molecular-weight fraction consistent with macroenzymemia [6]. Other specialized methods, such as gel filtration chromatography or immunofixation electrophoresis, can confirm macroenzyme detection when needed but are rarely required outside reference laboratories [6].

In this patient, severe but painless hyperlipasemia, normal CT imaging, very low dialysate amylase, and spontaneous downtrending of enzymes argued strongly against acute pancreatitis and prompted evaluation for macrolipasemia and macroamylasemia with PEG precipitation. Although PEG precipitation testing was requested to confirm macroenzymemia, the result was not available at the time of manuscript preparation; therefore, the diagnosis is based on the clinical, biochemical, and radiologic profile.

Role of dialysate enzyme analysis

In patients with peritoneal dialysis, dialysate enzyme levels provide important diagnostic context. In true acute pancreatitis, activated enzymes can leak from the inflamed pancreas into the peritoneal cavity, resulting in markedly elevated dialysate amylase levels, often in the hundreds or thousands of U/L [4,14]. High dialysate amylase in the setting of abdominal pain and elevated serum enzymes strongly suggests intra-abdominal pathology such as pancreatitis.

By contrast, in macroenzymemia or ESRD-related enzyme elevation without pancreatic inflammation, peritoneal dialysate enzyme levels typically remain low. Macroenzyme complexes (greater than 200 kDa) cannot traverse the peritoneal membrane, whereas monomeric enzymes (~50 kDa) can pass to some degree [6,14]. In this case, lipase greater than 4,000 U/L with dialysate amylase less than 30 U/L, combined with a normal pancreas on CT and absence of abdominal pain, strongly supported non-pancreatic hyperenzymemia rather than acute pancreatitis. Notably, this patient was not using icodextrin-based peritoneal dialysis solutions, which have been reported to influence serum enzyme measurements, further supporting the validity of the observed laboratory findings [15].

Diagnostic pitfalls and clinical implications

Conventional thresholds of lipase or amylase greater than three times ULN have good performance in the general population (sensitivity of 80-95% and specificity of 85-98%) but perform poorly in ESRD, where baseline enzyme elevation and macroenzymes are common [1,3,5]. Even extreme elevations, as in this case, may not indicate pancreatitis. Guidelines emphasize that diagnosis requires at least two of three criteria: characteristic abdominal pain, enzyme elevation greater than three times ULN, and imaging findings consistent with pancreatitis [1,2]. In ESRD, applying this framework requires careful attention to baseline enzyme values and clinical context.

Clinicians should suspect non-pancreatic hyperenzymemia in ESRD when: (1) enzyme elevations occur without abdominal pain, (2) enzyme patterns are atypical or persistently elevated, (3) imaging shows a normal pancreas despite very high enzymes, (4) peritoneal dialysate enzymes are low in PD patients, and (5) there are recurrent admissions for "pancreatitis" with consistently normal imaging.

Reflexively labeling any greater than three times ULN lipase elevation as "acute pancreatitis" can lead to unnecessary CT or MRI, prolonged NPO status, and an avoidable hospital stay [1,4]. In this patient, initial management as pancreatitis despite a benign abdominal exam contributed to a prolonged hospitalization before the possibility of macroenzymemia and ESRD-related hyperenzymemia was considered. His refractory gastroparesis, manifesting as nausea and vomiting, served as a red herring that initially distracted from the true cause of his enzyme elevations.

When macroenzymemia is suspected, PEG precipitation is an appropriate confirmatory study [6]. If macroenzymes are identified, this should be clearly documented in the medical record, and patients should be counseled that elevated enzyme levels do not always indicate pancreatitis. However, they remain at risk for genuine acute pancreatitis, which must still be diagnosed based on classic symptoms and imaging in addition to enzyme elevation, consistent with consensus criteria [1,2].

In ESRD patients presenting with abdominal symptoms, diagnostic algorithms should emphasize careful clinical assessment, serial enzyme measurements relative to baseline, imaging, peritoneal dialysate enzyme analysis (in PD patients), and macroenzyme testing when indicated, rather than relying solely on enzyme thresholds.

## Conclusions

This case illustrates the difficulty of interpreting markedly elevated pancreatic enzymes in ESRD. The patient's lipase exceeded 4,000 U/L, a level typically considered diagnostic of severe pancreatitis. Yet, he had no abdominal pain, normal pancreatic imaging, very low peritoneal dialysate amylase, and spontaneous enzyme downtrending without pancreas-specific therapy. These findings were most consistent with macrolipasemia and macroamylasemia in the setting of ESRD, rather than true pancreatitis.

Extreme hyperlipasemia can occur in dialysis patients without pancreatic disease, particularly when macroenzymes are present. Integrating clinical presentation, imaging, dialysis fluid analysis, enzyme trends, and targeted macroenzyme testing is essential to avoid misdiagnosis and unnecessary interventions. Considering macroenzyme-associated hyperlipasemia in the differential diagnosis for ESRD patients with elevated pancreatic enzymes allows more accurate diagnosis, more appropriate management, and better alignment of therapy with the underlying pathology.
